# CTCF driven TERRA transcription facilitates completion of telomere DNA replication

**DOI:** 10.1038/s41467-017-02212-w

**Published:** 2017-12-13

**Authors:** Kate Beishline, Olga Vladimirova, Stephen Tutton, Zhuo Wang, Zhong Deng, Paul M. Lieberman

**Affiliations:** 1 The Wistar Institute, Philadelphia, PA 19104 USA; 2 Present Address: Department of Biology and Allied Health Sciences, Bloomsburg University, Bloomsburg, PA 17815 USA

## Abstract

Telomere repeat DNA forms a nucleo-protein structure that can obstruct chromosomal DNA replication, especially under conditions of replication stress. Transcription of telomere repeats can initiate at subtelomeric CTCF-binding sites to generate telomere repeat-encoding RNA (TERRA), but the role of transcription, CTCF, and TERRA in telomere replication is not known. Here, we have used CRISPR/Cas9 gene editing to mutate CTCF-binding sites at the putative start site of TERRA transcripts for a class of subtelomeres. Under replication stress, telomeres lacking CTCF-driven TERRA exhibit sister-telomere loss and upon entry into mitosis, exhibit the formation of ultra-fine anaphase bridges and micronuclei. Importantly, these phenotypes could be rescued by the forced transcription of TERRA independent of CTCF binding. Our findings indicate that subtelomeric CTCF facilitates telomeric DNA replication by promoting TERRA transcription. Our findings also demonstrate that CTCF-driven TERRA transcription acts in *cis* to facilitate telomere repeat replication and chromosome stability.

## Abstract (web summary)

TERRA RNA is involved in maintaining stability during telomere repeat replication. Here the authors, by using CRISPR/Cas9, mutate CTCF-binding sites at start site of TERRA transcripts and find that subtelomeric CTCF facilitates telomeric DNA replication by promoting TERRA transcription.

## Introduction

Replication and maintenance of telomere repeat DNA is a major challenge to rapidly dividing cells^1–3^. Telomere repeats are bound by a multi-factor complex known as Shelterin that binds and protects telomeres throughout the cell cycle and maintains the double-strand and single-strand portions of the repeat sequences^4, 5^. Shelterins have well-characterized roles in regulating telomere repeat length homeostasis and DNA damage signaling. Adjacent to the TTAGGG tandem repeats are variable repetitive regions known as the subtelomeres^6, 7^. Subtelomeric sequences are bound by various nuclear factors that have been implicated in the regulation of transcription and chromatin structures important for telomere protection and regulation^8–12^.

One major function of the subtelomere sequences is to regulate the transcription of the telomere repeat-encoding RNA (TERRA). TERRA transcripts have been identified in multiple organisms, including yeast and human, and are known to initiate within subtelomeric repeat regions and proceed into the terminal (TTAGGG)_*n*_ repeats^13–15^. The function of these transcripts has been addressed in multiple studies, but it remains unclear whether these transcripts function directly on the telomeres from which they are transcribed (*cis*-acting) or whether they work remotely (*trans*-acting) on other telomeres or locations. Studies suggest that TERRA can bind both in *cis* to the telomere from which it is produced^16, 17^ as well as from a cellular pool that can interact in *trans* with other TERRA producing or non-producing chromosome ends^18, 19^. Transcribed TERRA has been shown to interact with the telomere DNA either through direct interaction and the formation of R-loops, or through interaction with RNA binding factors which recruit TERRA to telomeric chromatin^16, 20–26^. Other studies have shown that TERRA transcript levels peak during S phase, and further decrease as cells enter G2/M phase, suggesting that TERRA may function during cell replication^27, 28^. Additionally, TERRA is highly overexpressed in cancer cells utilizing alternative lengthening of telomere (ALT) mechanisms, which lack telomerase, but have variable length telomere repeats replicated in part through homologous recombination^16, 21, 24, 29–31^. All in all, the function of telomeric transcription and TERRA RNA in regulating telomere DNA replication, and whether it functions locally in *cis* or remotely in *trans*, remains unclear.

TERRA transcription initiates within the subtelomere and various regulatory elements have been implicated in its transcriptional control. The precise initiation site may vary for each chromosome, and a single telomere may be responsible for generating the majority of TERRA transcripts in some cells or organisms^19, 25^. A major initiating element for human TERRA was localized to a subtelomeric CpG-island^32^, which was found to contain high-affinity binding sites for CTCF and cohesin^9^. ChIP-Seq analyses also revealed that RNA polymerase II was enriched at these CTCF-cohesin sites in a large percentage of human subtelomeres^8, 9^. Depletion of CTCF and cohesin have resulted in alterations in TERRA transcription and telomere regulation, suggesting that TERRA transcription regulates telomere repeat stability. CTCF has many genomic functions, including the organization of higher-order 3D chromatin structures, nucleosome positioning, and regulation of RNA polymerase II initiation and RNA processing^33, 34^. It is yet unknown how the many possible genomic functions of CTCF are contributing to TERRA regulation and telomere maintenance. Additionally, more direct evidence that TERRA transcription contributes to the stability of the transcribed source telomere has not yet been provided. In this study we address the regulation and function for TERRA by CRISPR/CAS9 gene editing of a subtelomeric CTCF-binding motif implicated in TERRA transcription initiation. We show that CTCF-dependent transcription of the 17p subtelomere is important for completion of replication of that telomere under stress, and in the absence of CTCF and TERRA transcription, these sites form ultra-fine anaphase bridges and micronuclei during and after mitosis, respectively.

## Results

### CRISPR/Cas9 targeting of 17p family of subtelomeric CTCF-binding sites

**Fig. 1:**
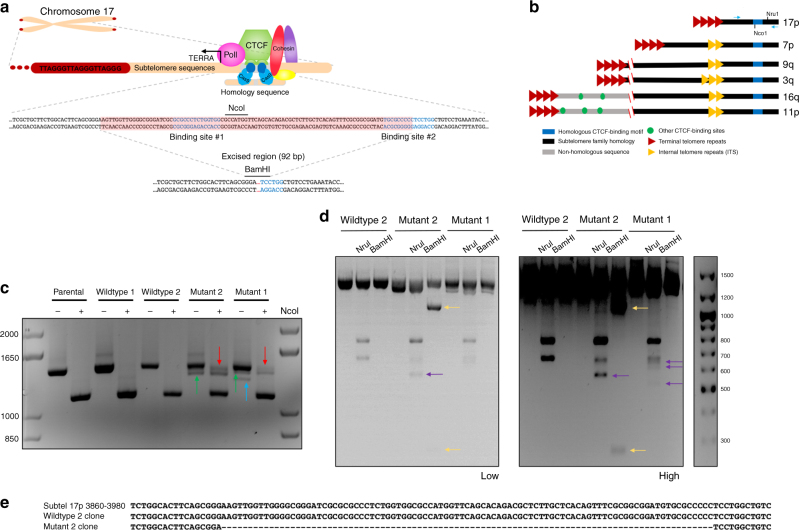
CRISPR-engineered mutants of CTCF 17p family subtelomere-binding sites. **a** CRISPR–Cas9 guide RNAs were designed to target regions around the CTCF-binding motif on the subtelomere of 17p. Homology-directed repair was promoted by co-transfection of a DNA fragment-containing homologous DNA sequences flanking a BamH1 site, created by deleting 92 bp base pairs of DNA surrounding the CTCF-binding motif. **b** Schematic depicting basic homology between 17p family of subtelomeres. **c** A 1400 bp fragment of DNA surrounding the deleted locus was PCR-amplified from genomic DNA collected from CRISPR clonal cell lines. PCR fragments from parental, or selected wildtype and mutant cells were undigested (−) or digested (+) with NcoI, a restriction site only present within the 92 bp deleted region. Undigested mutant fragments are indicated with red arrows. Green and blue arrows indicate mutant fragments of decreased size in undigested samples. DNA markers in first and last lanes. **d** Further digests with NruI and BamHI were utilized to identify mutant fragments. NruI digests outside the excised region only on 17p and 11p and generates two bands, 640/760 bp in wildtype and 548/760 bp in mutants. Purple arrows indicate bands created by mutant fragments. The BamHI cut site is introduced in clones that are repaired by perfect homologous recombination using the supplied gene block. Digestions creates 2 bands from mutant fragments, at 246 bp and 1063 bp, indicated by yellow arrows. Low (left) or high (right) image exposure. **e** Sanger DNA sequence validation of PCR fragment from Mutant 2 compared to wildtype clone and 17p consensus sequence

A series of highly conserved CTCF-binding motifs found on most subtelomeres within a few kilobases from the telomere repeats has been implicated in TERRA transcriptional regulation^9, 25^. We targeted the family of subtelomeric CTCF-binding sites represented by chromosome arm 17p, which is located in close proximity (<5 kb) to the (TTAGGG)_n_ repeat junction, shows strong colocalization with CTCF and cohesin in ChIP assays, and is known to produce TERRA in multiple cell types^8, 9^ (Fig. 1a). The 17p family CTCF-binding sites consist of a more extensive homology region (up to 20 kb) that is identical to that found at the terminal end of 7p, 9q, 3q, as well as non-terminal subtelomere repeat regions on 16q and 11p (Fig. 1b). The 17p family CTCF-binding sites are composed of 2 tandem CTCF-binding motifs, oriented in the direction of the telomere end, separated by about 35 bp of sequence. These CTCF sites are highly similar to those described previously to function at promoters and enhancers in transcriptional activation^35^. HCT116 cells were transfected with Cas9/gRNA constructs along with a homology block of DNA to promote repair of the chromosome target, replacing a 92 bp fragment around the CTCF-binding motifs with a single BamHI site. The cells were clonally selected and verified by PCR amplification, restriction digest, and sequencing (Fig. 1c, d). We selected two HCT116 clones, one containing a small fraction of mutated sites (Mutant 1), while the other containing a larger proportion of mutated sites (Mutant 2) among the multiple members of the 17p family of CTCF sites. Mutations in Mutant 2 could be validated by Sanger sequencing (Fig. 1e), and generated a single uniform PCR product with a BamHI restriction site insertion. Control cell lines were selected either from clonal selection of cells expressing no guide RNA (control for wildtype 1), or clones selected during the mutation validation stage, which were not found to contain any mutant copies (control for wildtype 2).

### CTCF-binding and H3K4me3 are lost at mutated CTCF sites

**Fig. 2:**
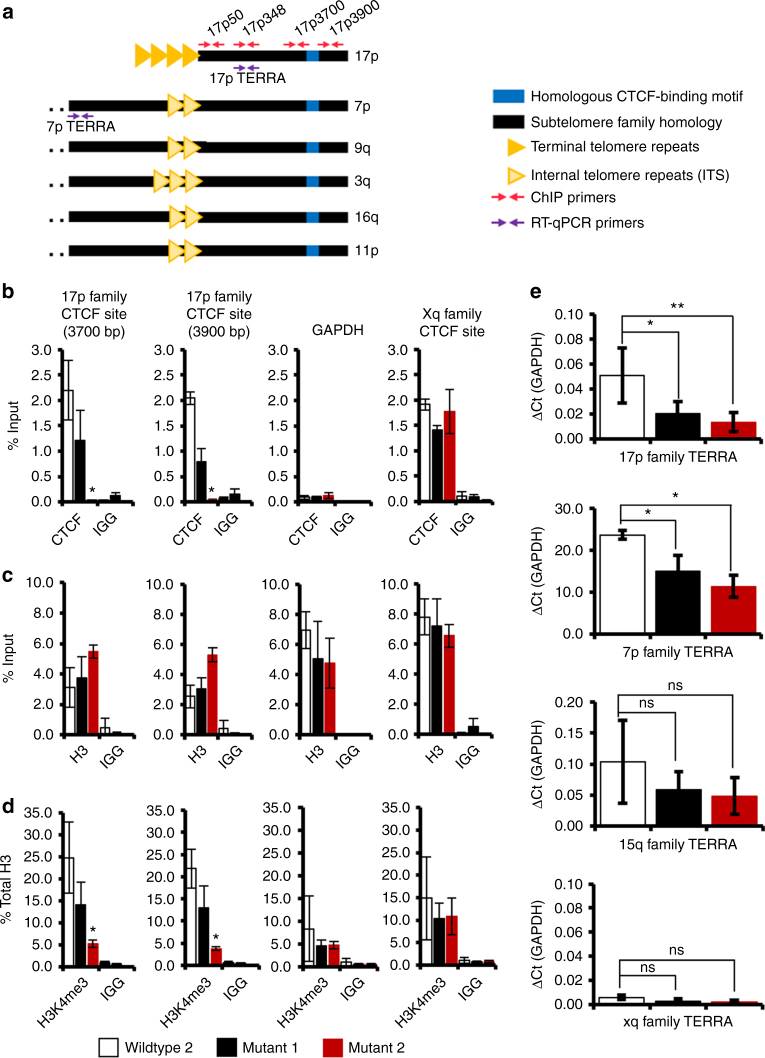
CTCF site-deletion mutants exhibit local decreases of subtelomeric CTCF, H3K4me3, and TERRA transcription. **a** Schematic of 17p family and 7p subfamily homology with position of primers used for RT-qPCR (purple) and ChIP-qPCR (red). **b** Wildtype (white bar) and mutant cells (black and red bars) were collected and chromatin immunoprecipitation was performed to look at binding of CTCF; **c** total histone H3; **d** histone H3K4me3 at either 17p family CTCF sites (primer set at 3700 bp relative or at 3900 bp relative to telomere repeat tracks), GAPDH, or the Xq family of subtelomeric CTCF sites. **e** RNA was isolated from cell lines and analyzed by RT-qPCR to assess chromosome-specific TERRA transcription levels for primers detecting all 17p family TERRA, 7p family, 15q family, or Xq family TERRA transcripts. All ChIP and RNA experiments represent 3–5 combined biological replicates with error bars expressing SEM of replicates. *p < 0.05; **p < 0.01, or not significant (ns), measured by Student's *t* test

CTCF binding in wildtype and mutant cell lines was verified by chromatin immunoprecipitation (ChIP) (Fig. 2a, b). The most extensively mutated cell line (Mutant 2) showed the largest decreases in CTCF binding around the mutated binding site, while it maintained CTCF binding at the Xq family of subtelomeres, which were not targeted by CRISPR mutation (Fig. 2b). Histone H3 binding around 17p family sites was maintained, with a modest trend toward an increase binding at regions adjacent to the deletions (Fig. 2c). In contrast, histone H3K4me3 levels were significantly decreased at sites adjacent to mutated CTCF-binding sites (Fig. 2d). Furthermore, mutant cells exhibited a decrease in TERRA expression of 17p family transcripts (including the 7p subfamily that contains mutated CTCF-binding sites), while other non-mutated chromosome TERRA transcripts (15q and Xq) and global levels of TERRA transcripts showed no significant change (Fig. 2e; Supplementary Fig. 1a, b). We did not detect any changes in TRF2 binding to adjacent subtelomeric sequences at 17p-mutated telomeres or at telomere repeat DNA (Supplementary Fig. 1c, d). These findings indicate that CRISPR/Cas9-mutated CTCF-binding sites on the 17p subtelomere family lead to a local loss of CTCF, histone H3K4me3, and decrease TERRA transcription, but do not disrupt global TERRA levels or TRF2 binding.

### 17p CTCF site mutants exhibit sister-telomere loss under replication stress

**Fig. 3:**
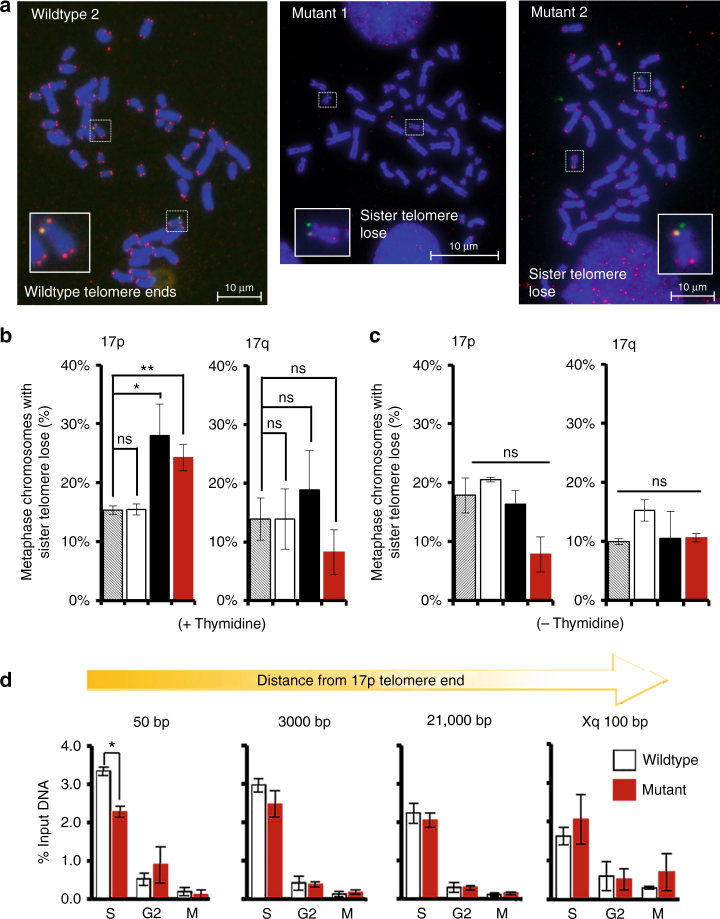
CTCF site mutant 17p telomere ends exhibit mitotic sister-telomere loss and decreased replication. **a** Cells were synchronized by double thymidine block, released for 7 h, colcemid was added, and mitotic cells were collected 1 h later. Representative images of mitotic spreads in wildtype 2, Mutant 1, or Mutant 2 clones stained with dual FISH for telomere repeats (red) and 17p subtelomeric sequences (green). Left, wildtype; Center, 17p Mutant 1; Right, 17p Mutant 2. **b**, **c** Quantification of sister-telomere loss on chromosome 17, p and q arms in cells synchronized with double thymidine block (+thymidine) (**b**), or asynchronous cells (−thymidine) (**c**). Data represented as average of 3 biological replicates, *n* = 150–200 metaphases, error bars represent SEM. *p < 0.05; **p < 0.01, measured by Student's *t* test. **d** Cells were synchronized and released in the presence of BrdU either during the first 6 h, for S phase or from 6–8 h for late S/G2, or from 7–9 h for M phase. Genomic DNA was isolated and BrdU incorporation was measured by immunoprecipitation with an anti-BrdU antibody. Data represents average of three independent experiments with error in SEM

We next determined the effect of CTCF-binding site mutation on telomere integrity. Southern blot telomere-length assays revealed that bulk telomere length for mutant 2 was slightly greater than wildtype clone and parental cells (Supplementary Fig. 1f, g). However, this loss may be due to clonal variation, as the telomere length remained stable and did not continue to shorten in mutant cell lines. Analysis of metaphase spreads from thymidine synchronized cells revealed a significant increase in sister-telomere loss in mutant cell lines (Fig. 3a–c). These effects were specifically enriched at 17p telomeres as determined by dual FISH using FISH probes specific for C-strand telomere repeat (red) and 17p subtelomeric DNA (green) (Fig. 3a, b). Sister-telomere signal loss was also observed using a G-strand telomere probe (Supplementary Fig. 2a), indicating that there is no strand specificity to sister-telomere loss. Sister-telomere loss was not observed at the distal 17q arm of mutant cell lines (Fig. 3b, right panel), indicating that the effect occurs proximal to the deletion of subtelomeric CTCF. We did not observe any significant differences in signal-free ends where both sister signals are lost (Supplementary Fig. 2b–d). Interestingly, the loss of sister-telomere signal was not observed in metaphase spreads from cells that were not pre-synchronized with double thymidine block (Fig. 3c). As double thymidine treatment can lead to replication stress, it is possible that subtelomeric CTCF facilitates sister-telomere replication especially under conditions of replication stress.

### 17p CTCF site mutants exhibit decreased replication under stress

To test whether mutation of subtelomeric CTCF-altered DNA replication, we measured the levels of BrdU incorporation by BrdU-immunoprecipitation assay at sites adjacent to subtelomeric region of 17p in wildtype vs. mutant cells. Cells were synchronized by double thymidine block and released, followed by incorporation of BrdU during S phase. Replicated DNA was assessed by BrdU IP followed by qPCR. Mutant cells showed a significant loss of BrdU incorporation during S phase at subtelomeric regions closest to the 17p family telomere repeats compared to wildtype cells (Fig. 3d), a difference that diminished with distance from the repeats. No difference in BrdU incorporation was seen at other telomere adjacent loci. Flow cytometry indicated that the cells cycled at the same rate after treatment (Supplementary Fig. 3a) and a time course of BrdU incorporation at the loci closest to the 17p telomere end as well as other subtelomere ends showed no significant change in the rate of BrdU incorporation between mutant and wildtype cells (Supplementary Fig. 3b). These data suggest that subtelomeric CTCF-binding site at 17p is required for the completion of local DNA replication, but does not change the overall rate or genome-wide DNA replication.

### 17p CTCF site mutants exhibit ultra-fine anaphase bridges

**Fig. 4:**
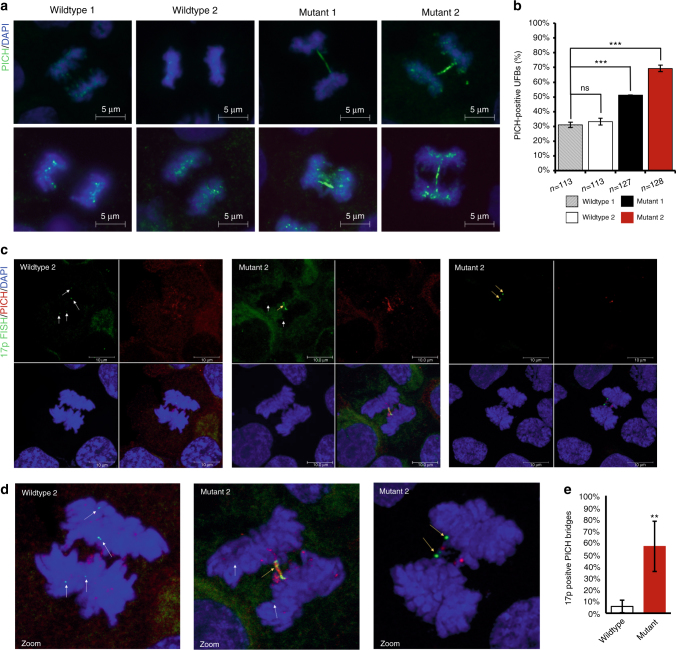
CTCF site mutant 17p telomere ends form ultra-fine anaphase bridges (UFBs). **a** Cells were synchronized by thymidine block and collected between 8–9 h post release and stained with PICH (green) to detect ultra-fine bridges in anaphase cells. **b** Quantification of **a**. **c** Cells were treated as in **b** and co-stained for PICH protein and 17p chromosome FISH. Representative image of anaphase bridges for wildtype and Mutant 2. White arrows indicate unaffected 17p FISH signals; yellow arrows indicate 17p FISH signals co-localizing with PICH-positive UFBs. **d** zoomed fields of merged images in **c**. **e** Quantification of **c**. IF/FISH experiments represent average of three biological replicates and SEM; **p < 0.01; ***p < 0.001, measured by Student's *t* test

Several studies demonstrate that replication stress can lead to the formation of ultra-fine anaphase bridges^36, 37^. Ultra-fine anaphase bridges (UFBs) are thought to form due to the presence of un-replicated regions or those which have not been decatenated prior to anaphase^36, 38^. To determine whether telomeres lacking subtelomeric CTCF-binding sites formed ultra-fine anaphase bridges, we analyzed the CTCF-deleted chromosome arm at 17p through metaphase and into anaphase. Anaphase preparations of synchronized cells were stained for PLK1-interacting checkpoint helicase (PICH), a DNA helicase known to bind to ultra-fine anaphase bridges^38^. We found mutant cell lines had an increased number of PICH-positive UFBs during mitosis (Fig. 4a, b; Supplementary Fig. 4a). When we co-stained anaphase cells with PICH and 17p FISH, we found a significant enrichment of the 17p chromosome arm on or flanking PICH bridges in mutant cells while 17p presence near PICH bridges in wildtype cells was extremely rare (Fig. 4c–e; Supplementary Fig. 4a). These findings suggest that subtelomeric CTCF functions locally and in *cis* to facilitate the completion of telomere DNA replication.

Sister-telomere loss in the presence of thymidine block, as well as formation of UFBs at the 17p telomere end suggested a possible sensitivity of the mutant cells to replication stress. We treated cells with another common replication stress-inducing agent aphidicolin to test for a similar sensitivity. We saw similar trends in UFB formation in aphidicolin-treated mutant cells as those we saw with thymidine treatment (Supplementary Fig. 4b,c). On the other hand, aphidicolin treatment did not induce the same extent of sister-telomere loss as was observed with double thymidine block (Supplementary Fig. 4d), perhaps because the thymidine treated cells go through S phase stress twice while aphidicolin is only a single-cell cycle without passage through mitosis. Nevertheless, these findings support the model that subtelomeric CTCF is important for overcoming different forms of replication stress at adjacent telomere repeat DNA.

### Increased TERRA transcription rescues defects in 17p CTCF site mutants

**Fig. 5:**
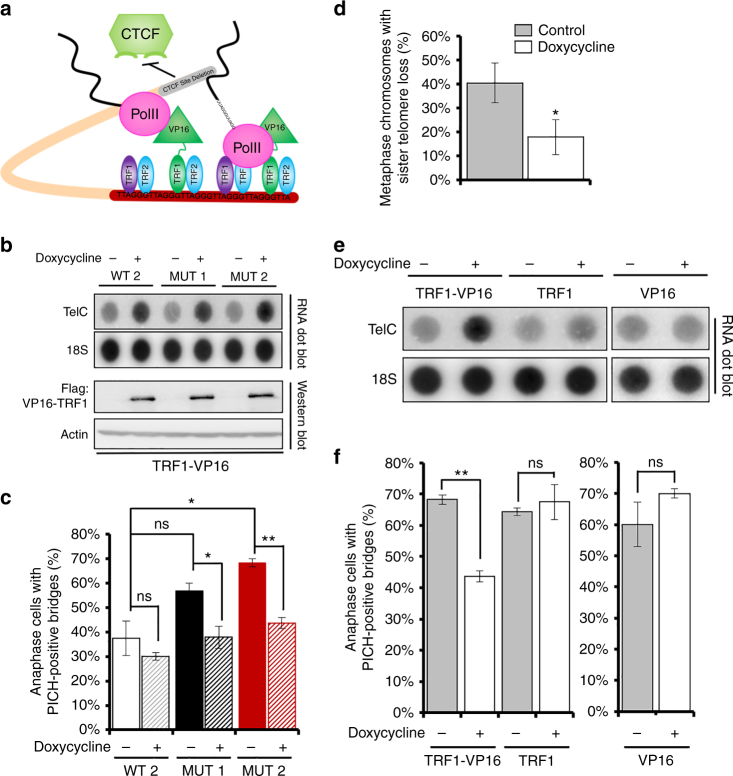
Increased TERRA transcription rescues mitotic defects in CTCF site mutant cells. Stable mutant and wildtype cell lines expressing a TET-inducible fusion of TRF1–VP16 were selected. Cells were synchronized and expression of the fusion protein was induced 12 h prior to thymidine release. Cells were collected 8–9 h post release and processed to measure TERRA expression and ultra-fine anaphase bridge formation. **a** Cartoon of VP16–TRF1-mediated rescue of TERRA transcription. **b** DotBlot of RNA collected after doxycycline induction in cells expressing the TRF1–VP16 construct in wildtype (WT2), Mutant 1 (Mut 1), or Mutant 2 (Mut 2) cells probed for TelC or 18S (top panels). Western blot of TRF1–VP16 and Actin control for same samples (lowerpanels). **c** Quantification of percentage of cells with PICH-positive anaphase bridges after treatment shown in **b**. **d** Mutant 2 cells treated as in **b **were treated with colcemid and collected for metaphase spreads. Sister-telomere loss was quantified on 17p. **e** Mutant 2 cells expressing inducible TRF1–VP16 fusion were compared to those expressing TRF1 only or VP16 only by RNA dotblot for TERRA and 18S. **f** UFBs were quantified for cells treated as described in **e**

To test whether the telomere replication defects were due to CTCF regulation of TERRA transcription and not another CTCF function at the subtelomere, we generated wildtype and mutant clones that could induce TERRA transcription independently of CTCF. Previous studies have shown that fusing TRF1 to the strong transcriptional activation domain of the HSV1 VP16 protein could induce high levels of TERRA^39^. Therefore, we generated wildtype and mutant clones expressing an inducible TRF1–VP16 fusion protein (Fig. 5a). Doxycycline induction of TRF1–VP16 led to a rapid increase in TERRA transcripts in wildtype and mutant cells (Fig. 5b). Remarkably, doxycycline induction of TERRA reduced the appearance of ultra-fine anaphase bridges (Fig. 5c), and reduced sister-telomere loss in mutant CTCF site lines (Fig. 5d). Induction of TRF1 lacking VP16 or VP16 alone in mutant cells did not induce TERRA expression (Fig. 5e), and did not reduce the number of ultra-fine anaphase bridges (Fig. 5f). These findings indicate that transcriptional activation of TERRA can overcome telomere-specific defects associated with conditions of replication stress.

### 17p CTCF site mutants exhibit decreased viability and genomic instability

**Fig. 6:**
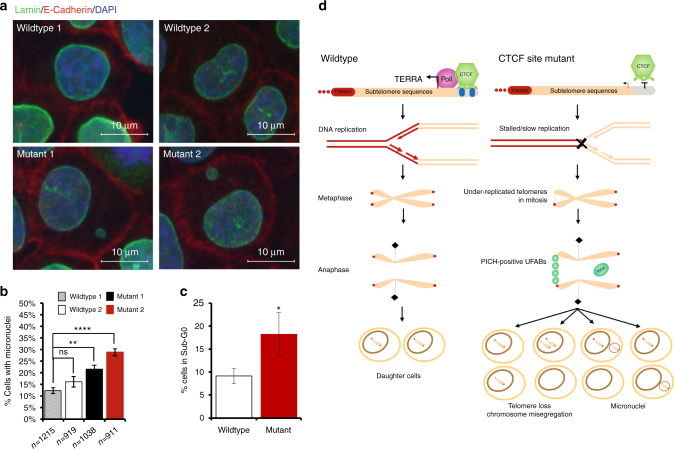
CTCF site mutant cells exhibit post-mitotic micronuclei and decreased viability. **a** Wildtype 1 and 2, or Mutant 1 and 2 cells were arrested with thymidine followed by release for 24 h and then stained with Lamin A/C (nuclear membrane) and E-Cadherin (cell membrane) to assess micronuclei formation after mitosis. Representative images of merged channels for each cell type. **b** Quantification of **a**. All IF/FISH experiments represent average of at least 3 biological replicates and SEM. *p < 0.05, **p < 0.01, and ****p < 0.0001, measured by Student *t* test. **c** Cells for wildtype 2 and Mutant 2 were collected 24 h after thymidine release and assessed by flow cytometry for sub-G0 cell population. Data represents average of three independent biological replications and SEM, *p < 0.05, by *t* test. **d** Schematic depicting effect of CTCF-mediated TERRA transcriptional on telomere replication and mitosis

We next tested whether these cell cycle defects lead to any observable phenotypes after cell division. Cells treated with thymidine were analyzed by microscopy at 24 h after release, ~12 h after mitosis. We found that mutant cells exhibited an increased level of micronuclei relative to wildtype cells (Fig. 6a, b). Flow cytometry revealed a corresponding increase in the sub-G0 cell population after thymidine treatment (Fig. 6c), or aphidicolin (Supplementary Fig. 5a) in mutant, but not wildtype cells. Additionally, treatment with replication stress-inducing agents (e.g., thymidine or aphidicolin) induced an increased level of the DNA damage marker γH2Ax and phospho-p53 in mutant cells similar to wildtype (Supplementary Fig. 5b, c). This indicates that double thymidine block and aphidicolin treatment both induce a persistent DNA damage consistent with the induction of replication stress. Furthermore, mutation of subtelomeric CTCF leads to survivors that exhibit genomic abnormalities, such as micronuclei formation and increased population of SubG1 cells.

## Discussion

Our findings implicate subtelomeric CTCF and TERRA transcription in the proper maintenance of adjacent telomere repeat DNA. CRISPR/Cas9 mutation of the CTCF regulatory element controlling TERRA reveals a potential function for TERRA transcription in promoting the completion of DNA replication at the telomere ends (Fig. 6d, left). Cells lacking subtelomeric CTCF-binding sites were sensitive to replicative stress resulting in mitotic dysfunction with loss of sister-telomere repeat signal and the formation of ultra-fine anaphase bridges (Fig. 6d, right). This sensitivity to replicative stress persists in mutant cells through the rest of the cell cycle and causes decreased viability of sister cells and the formation of micronuclei. Mechanistically, we show that ectopic expression of VP16–TRF1, which increases TERRA transcription, can protect against the formation of PICH-positive anaphase bridges. We propose that CTCF-driven TERRA transcription initiating within the subtelomere facilitates the completion of DNA replication of adjacent telomere repeats.

TERRA transcription may enhance telomere maintenance by overcoming some of many telomere-specific challenges to DNA replication^40^. Telomere transcription and TERRA molecules may facilitate DNA replication by promoting the dissociation of shelterin components that may otherwise obstruct replication fork progression^41^. Telomeres typically replicate unidirectionally^42^ and late in the cell cycle^43, 44^ and cell cycle delays due to replication stress are likely to lead to a loss of telomere repeat DNA. TERRA transcription may advance the replication timing of telomere repeat regions^45^, and therefore increase the probability of completing DNA replication. Thus, TERRA transcription may facilitate telomere DNA replication by multiple mechanisms.

TERRA may also have complex regulatory functions at telomeres. TERRA has been implicated in the formation of R-loops^23^, and G-quadruplex structures in the telomere repeats^46^, structures which may inhibit, as well as promote telomere DNA replication. R-loop structures may act as a barrier to replication machinery^20, 22^, although there are normal cell pathways that promote bypass of R-loop structures. In contrast, G-quadruplex structures, suggested to form on the opposing strand of the R-loop, have been shown to support origin binding and replication initiation, which could be utilized as a means to initiate replication from telomere repeats or rescue a stalled fork heading into the repeats from a subtelomeric origin^47, 48^.

In addition to TERRA transcription, CTCF may facilitate telomere DNA replication by altering chromatin structure. The CTCF-binding site is required for active histone H3K4me3 enrichment (Fig. 2), and may prevent the formation of heterochromatin that could otherwise further impede DNA replication through telomeres. The 17p family of subtelomeric CTCF-binding motifs have structural similarity to the tandem copies that have been implicated in transcriptional regulatory function^35^. These type of motifs have been found to be enriched at other repetitive elements^49^, suggesting that CTCF plays an important regulatory role at repetitive elements. CTCF-binding sites show significant genetic variation in tumor tissues, and some of these variations have been implicated in altered transcription control in cancer cells^50–53^. However, we have been unable to identify any significant variations in the 17p family of subtelomeric CTCF-binding sites from the TCGA database or 1000 genomes, suggesting that these CTCF sites may be essential for human cell viability.

Finally, it has been suggested that the majority of TERRA transcripts arise from a single subtelomere and interact in *trans* with all the telomere ends^18^. Our data does not exclude the possibility that large quantities of TERRA are generated from a single chromosome in some cell types, nor that TERRA transcripts can interact in *trans* with other telomere ends. However, our data strongly suggest that CTCF-binding site mutation and decreased levels of TERRA transcription directly impacts the DNA replication efficiency of the same source telomere end from which it is transcribed, indicating that it has a direct *cis*-acting effect on telomere stability. In conclusion, the data presented here supports a function for CTCF-mediated TERRA transcription in maintaining proper telomere replication and chromosome stability.

## Methods

### Cell culture and treatments

HCT116 cells (purchased from ATCC) were cultured in DMEM with 10% FBS and penicillin/streptomycin. Clones expressing VP16 constructs were cultured in 10% tet-free FBS (Sigma) and G-418. Cells were in 2.5 mM thymidine and 0.2 μM aphidicolin, and mitotic cells were collected in 0.1 μg/mL colcemid. For aphidicolin experiments and BrdU-immunoprecipitation assays, Cdk1 inhibitor, RO-3306, was used at a dose of 9 μM to inhibit cells from entering mitosis. For BrdU-immunoprecipitation assays and BrdU was added at 10 μM at indicated time points.

### Vectors and cloning

Cas9/guide RNA dual vectors were constructed using sequences for 17p-targeting gRNA (Supplementary Table 3)^54^. Homology blocks were constructed from subtelomere sequences and synthesized by IDT Inc, sequences are also listed in Supplementary Table 4. To construct the TRF1–VP16 constructs, TRF1ΔN (44–439), and VP16–TRF1ΔN (44–439) were sub-cloned into the pINDUCER20 vector^55^ from plasmids for TERRA induction^39^. Briefly, the DNA fragments were introduced into the pENTR Directional TOPO Cloning vector (Invitrogen) as per the manufacturer’s instructions, and followed by the Gateway cloning into the pINDUCER20 vector using the LR Clonase II (Invitrogen).

### CRISPR cell-line construction

Homology blocks were amplified by PCR and co-transfected into cells with Cas9 constructs. Cells were clonally selected with puromycin for 10–14 days. Genomic DNA was isolated from all clones, and mutations were validated by PCR and restriction digest. TRF1–VP16 lines were constructed by lentiviral introduction of vectors. Lentivirus was produced from 293T cells by co-transfecting the constructs with viral packaging vectors pVSVG, pRSV, and pMDL. All the cultured media were supplemented with 10% (vol/vol) tetracyclin-free FBS (Corning) and harvested at 48 h after transfection. To generate the inducible cell lines, 1 × 10^6^ HCT116 cells were infected with 1 mL lentivirus overnight in the presence of 2 μg/mL Polybrene (Sigma). Infected cells were selected by 1 mg/mL G418 48 h after infection. Cells were stably selected for 6 days in the tetracyclin-free medium prior to use in experiments. For TERRA induction, 0.5 µg/mL Doxycycline was added to the medium 12 h prior to thymidine release in synchronized experiments.

### Chromatin immunoprecipitation

Cells were fixed and processed for chromatin immunoprecipitation^9^. Briefly, cells were fixed with formaldehyde for 15 min, washed, and lysed in SDS Lysis buffer. Chromatin lysates were sonicated for five cycles, 10 min each, 30 s on/off. Lysates were diluted and pre-cleared with protein A sepharose and bacterial tRNA for blocking. Lysates were immunoprecipitated with indicated antibodies overnight. Antibodies (2 μg of each) used were as follows: anti-CTCF, EMD Millipore 07-729; anti-H3, Active Motif #39163; and anti-H3K4me3, EMD Millipore 07-473. Immune complexes were collected with protein A sepharose for 2 h, beads were washed, and bound DNA was collected, and then purified with PCR purification kit (Sigma). Protein binding was determined by qPCR or DNA dotblot. Primers used in qPCR assessment are listed in Supplementary Table 1 and locations of 17p primers are shown visually in Supplementary Fig. 1a.

### RT-qPCR and RNA DotBlot

RNA was isolated using Trizol (Invitrogen) following manufacture's instructions. An aliquot of 5 μg of RNA was blotted onto membrane for dot blots^9^. RNA was DNase treated and converted to random primed cDNA using Superscript III kit (Invitrogen). cDNA was assessed for TERRA transcription levels, using GAPDH as control. Primers for qPCR analysis are listed in Supplementary Table 2 and locations of 17p primers are shown visually in Supplementary Fig. 1a.

### Brdu immunoprecipitation

Genomic DNA was collected from cells after incorporation of BrdU. DNA isolation kit (Promega). DNA was sonicated to between 100–500 bp in length. Sonicated DNA was denatured for 5 min at 100 C and diluted in IP buffer (10 mM Sodium Phosphate pH7, 0.14 M NaCl, 0.05% Triton X-100). BrdU containing DNA was immunoprecipitated with 2–4 µg of anti-BrdU antibody (BD Pharmingin mouse-anti BrdU Clone 44 (#347850)) for 1 h at room temperature and collected using protein G agarose beads for 30 min. Isolated DNA/immune complexes were washed 4 times with IP buffer and dissociated using BrdU IP lysis buffer (10 mM EDTA, 20 mM Tris pH 8.8, 0.5% SDS, 0.25 mg/mL Proteinase K) overnight. DNA was purified by column purification (Sigma). BrdU incorporation into genomic regions was assessed by qPCR.

### Immunofluorescence and DNA-FISH

For non-metaphase cell preparations, cells were collected by trypsinization and counted. 1 × 10^5^ cells were spun onto 10 mm coverslips for processing. Fixation was done with either 1% PFA for immunofluorescence only or 50:50 mix methanol:acetone for IF–FISH. IF-only cells were extracted with 0.5% NP-40 for 10 min and probed with primary (1:300) and secondary antibodies (1:500). Primary antibodies for PICH were from Abnova (H00054821-B01P) or EMD Millipore (04-1540); E-Cadherin, Abcam (ab53033); and Lamin A/C, DSHB (MANLAC1(4A7). Secondary Antibodies; Goat anti-mouse/anti-rabbit AlexaFluor-488 (Thermo Fisher, A-11029 and A-1134) and AlexaFluor-594 (Thermo Fisher, A-11032 and A-11037) were used. Cells were counterstained with DAPI and imaged using a Leica DMRE upright microscope.

IF–FISH cells were stained with primary and secondary antibodies after fixation and then post-fixed with 2%PFA + 1%TritonX100. The primary antibodies for PICH used was anti-PICH, Abnova (H00054821-B01P, 1:100). Secondary anti-mouse AlexaFluor-594 (Thermo Fisher, A-11032, 1:500). Cells were washed with PBS, then extracted with 0.2 N HCL/0.02%TritonX100 for 10 min at 4 C. The cells were dehydrated with ethanol series and probed with FISH probe (Cytocell LPT17pG) overnight at 37 C. Coverslips were washed 2× with Wash 1 (70% Formamide, 10 mM Tris pH7–7.5, 0.1% BSA), 3× Wash 2 (0.1 M Tris pH7–7.5, 0.15 M NaCl, 0.08%Tween), counterstained with DAPI, and mounted on slides for imaging. The slides were imaged with Leica TCS SP5 II scanning laser confocal microscope. Anaphase cells were imaged as a Z stack and representative images are Maximum Projections of all stacks.

Mitotic cells were collected by trypsinization and swelled in 0.025 M KCl for 25 min at 37 C. Cells were fixed with 3 resuspensions of a methanol: acetic acid, 3:1 mix, and stored in fixative at least 24 h prior to dropping. Mitotic cells were dropped onto chilled clean glass slides and dried at least 4 h prior to staining. After drying, the cells were fixed for 5 min with 1% paraformaldehyde in PBS. Cells were washed and then dehydrated with ethanol wash series. Cells were co-stained with 17p subtelomere probe (Cytocell LPT17pG or LPT17pR) and telomere repeat (CCCTAA) PNA probe, TelC-TMR or telomere repeat (GGGATT) PNA probe, and TelG-FAM (Panagene) in Hybridization Buffer B (Cytocell) overnight at 37 C. Slides were washed as described above with Wash 1 and 2, and counterstained with DAPI. Slides were dehydrated with another ethanol series and mounted with Slow Anti Fade Gold mounting solution (Invitrogen). Mounted Slides were imaged on Leica DMRE upright microscope.

### Data availability

All data presented in this manuscript is available from the authors.

## Electronic supplementary material

Supplementary Information
