# Supplementary material for: CTCF driven TERRA transcription facilitates completion of telomere DNA replication
Source: Nat Commun. 2017 Dec 13;8:2114. doi: 10.1038/s41467-017-02212-w (PMC5727389; doi:10.1038/s41467-017-02212-w)
Supplement: Supplementary file 1 — Supplementary Information [file 41467_2017_2212_MOESM1_ESM.pdf]

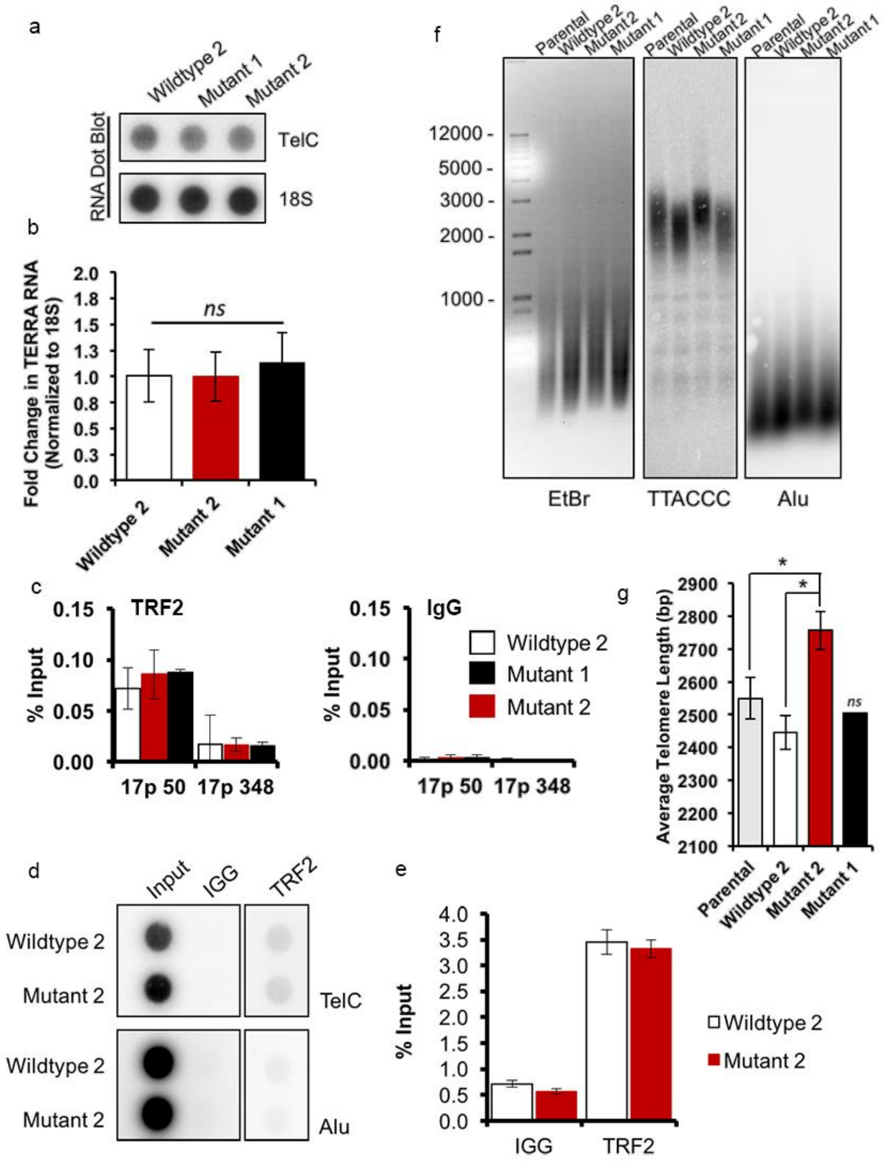

Supplementary Figure 1: TRF2 binding and telomere length are unaffected in asynchronously growing CTCF site mutant cells. a, Representative RNA Dotblot showing total TERRA levels in mutant cells lines compared to wildtype control. **b**, Quantification of RNA Dotblots represented in panel **a.** **c,** Chromatin Immunoprecipitation analysis of TRF2 binding. ChIP-qPCR for TRF2 (left) or IgG control (right) analyzed for binding at 17p subtelomere with primers situated 50 or 348 bp from telomere repeat track. **d,** ChIP Dot blot assay of TRF2 or IgG control for binding telomere repeat DNA (top panel) or Alu DNA (lower panel). **e,** Quantification of representative ChIP-Dot blots shown in panel **d**. **f,** Southern blot analysis of telomere length of wildtype and mutant CRISPR clones. **g,** Average telomere length of wildtype and mutant cell cells, bars represent an average of 3 independent DNA isolations and Southern blots, error bars represent SEM.

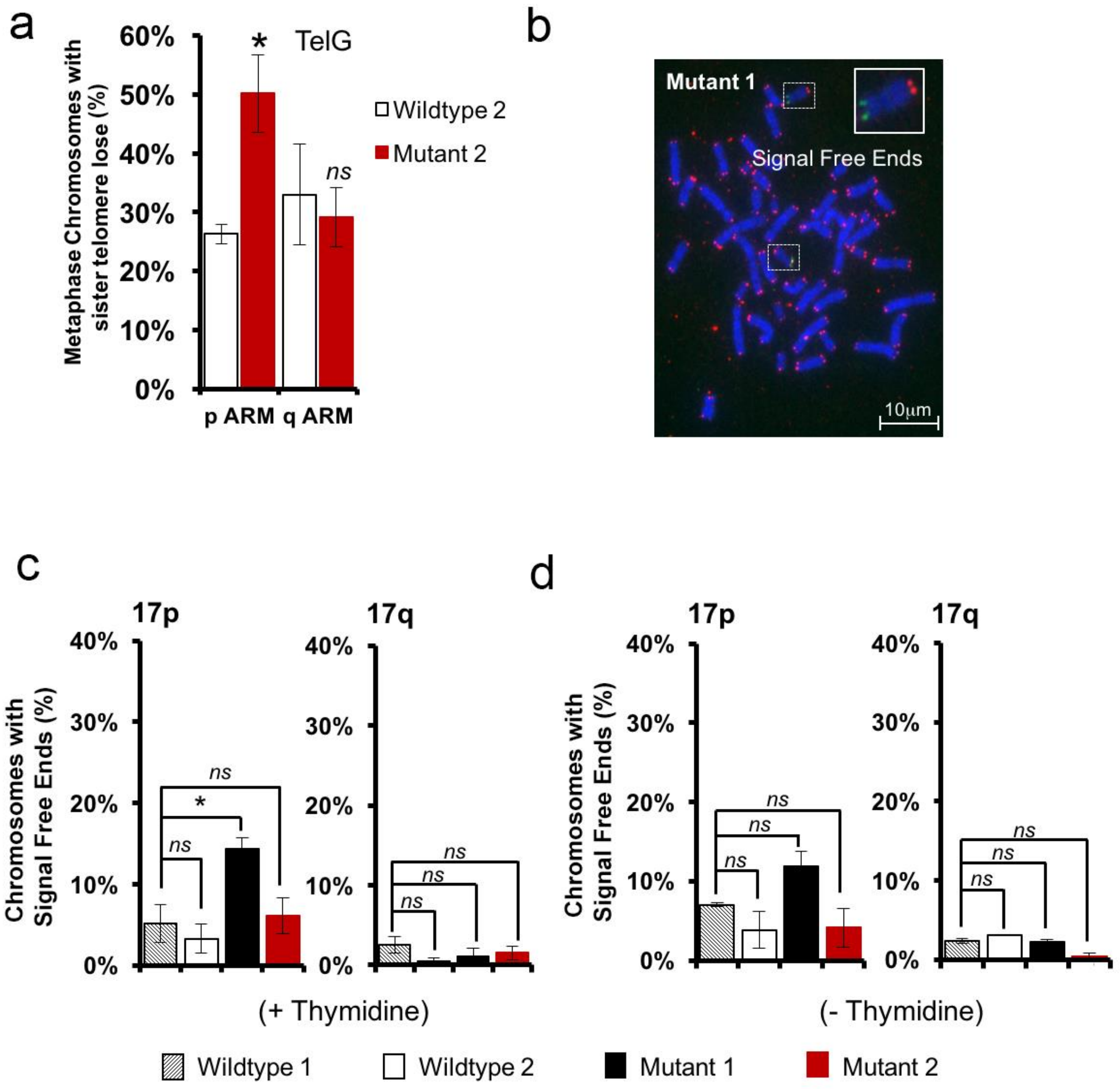

Supplementary Figure 2: Asynchronous cells exhibit no defect in sister telomere loss or signal free ends. **a,** Quantification of sister telomere loss as described in Figure 2a,b except using the TelG PNA probe to analyze C-rich strand of telomere repeat DNA for Wildtype 2 (white) and Mutant 2 (red). **b**, representative image of signal free ends. **c,** Quantification of signal free ends in cell synchronized with thymidine as described for Fig. 3b. **d**, Quantification of signal free ends in asynchronous cells (-thymidine) as described for Fig. 3c. Bars represent average 3 experiments, error represented as SEM.

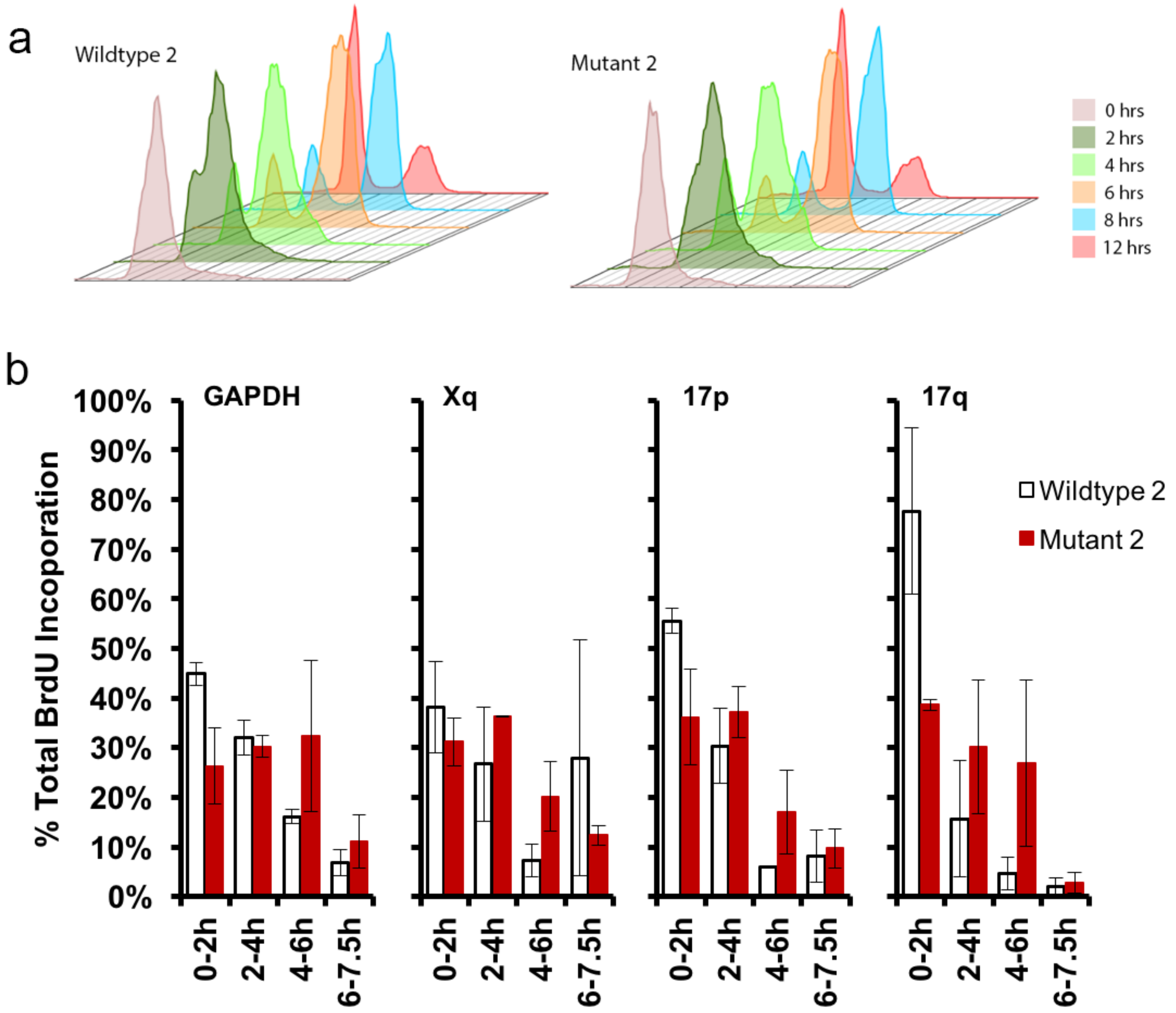

Supplementary Figure 3: a, Cell cycle profiles of wildtype and mutant cells released from double thymidine block and collected at indicated time points. **b,** Cells were prepared in Fig. 3d but BrdU was pulsed for the indicated time frames on x-axis. All cells were collected after 7.5 hrs and isolated DNA was used for IP. Total percentage of BrdU at each time point was calculated from total incorporation over the whole time course. Data represents average of 3 independent experiments with error in SEM.

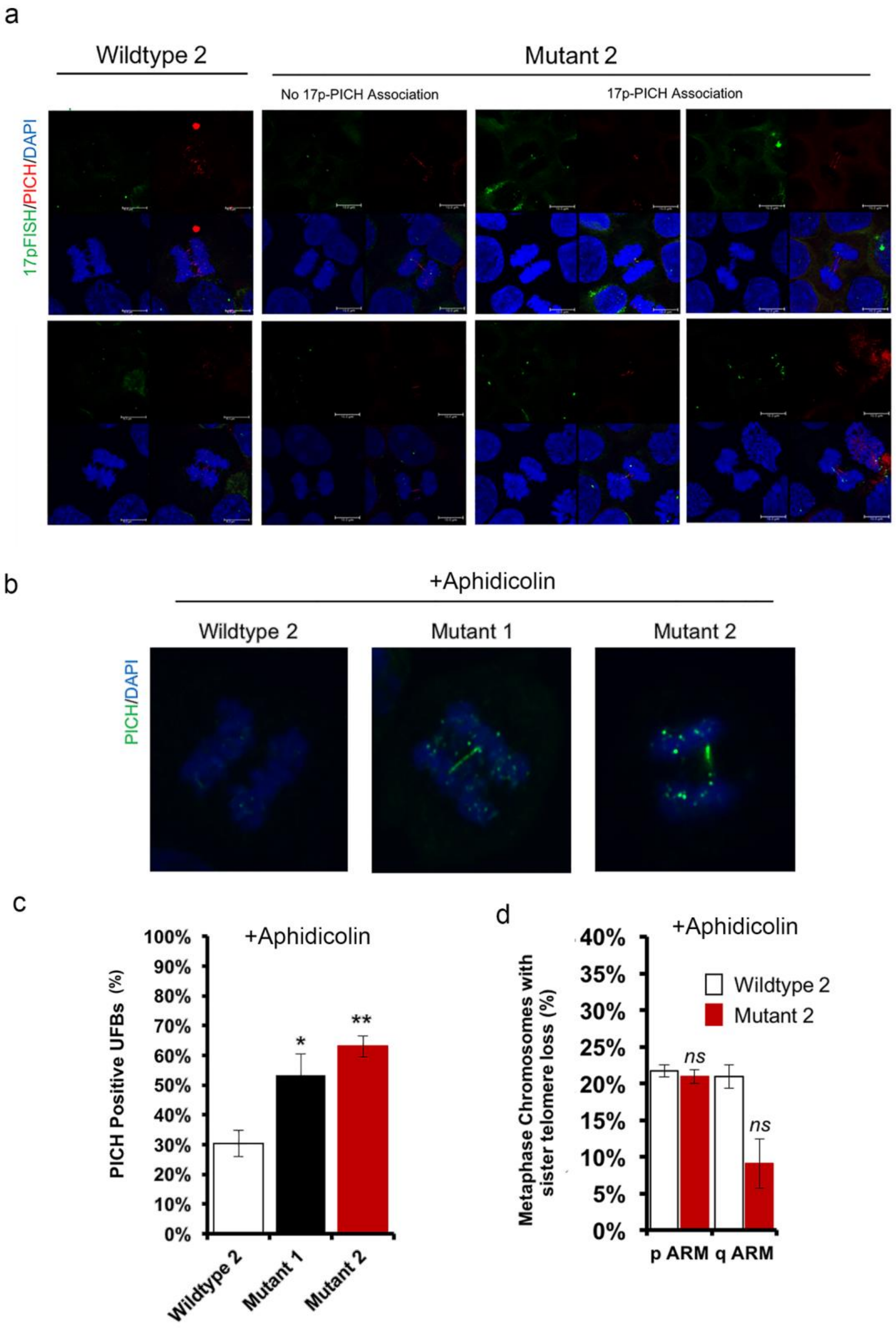

Supplementary Figure 4: CTCF site mutant 17p telomere ends form ultra-fine anaphase bridges. a, Additional images of cells from Figure 4c. **b,** Cells were treated with aphidicolin for approximately 18hrs in the presence of cdk inhibitor RO-3306 to collect at the G2/M border. Cells were released into mitosis and collected approximately 1hr post release and assessed for UFB formation. **c**, Quantification of cells stained in **b**. **d,** Quantification of % of metaphase chromosomes with sister telomere loss in cells treated as in panel **b**.

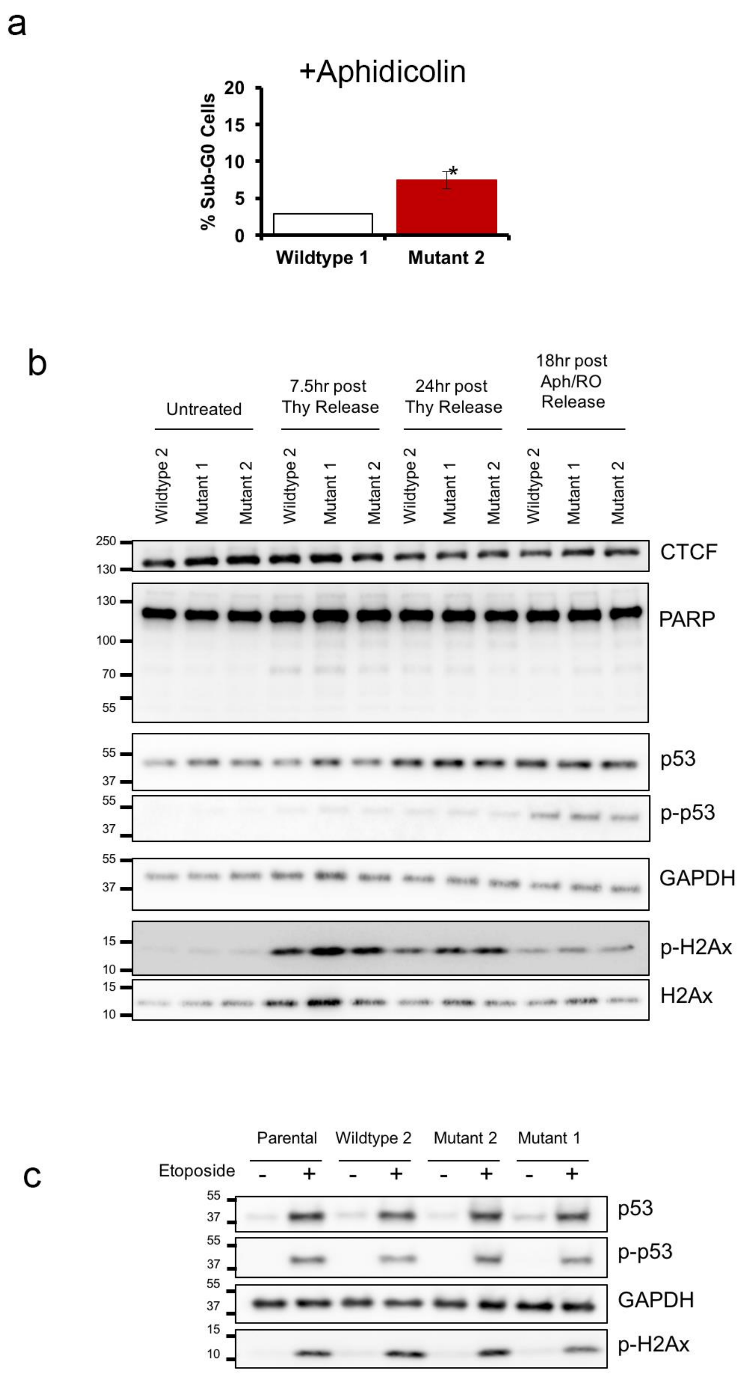

Supplementary Figure 5: Mutant cells exhibit increased DNA damage in response to replication stress. a, Cells were collected 24 hrs after release from aphidicolin/RO-3306 treatment and assessed for sub-G0 cell population. Data represents average 3 independent experiments with bars representing SEM. **b**, Protein was collected from thymidine synchronized or aphidicolin treated cells at various time points and assessed by Western blot for the indicated proteins. **c**, Wildtype and mutant cells were treated with etoposide at collected 24hrs after treatment, protein was collected and assessed by Western blot for indicated proteins.

**SupplementaryTable 1: ChIP Primers**		
Name	Sequence	Target Sequence
17p 3700 fwd	CTCACCAATGAAGAACACCAAATTA	17p3789-3813
17p 3700 rev	GCTGAAGTGCCAGAAGCA	17p3856-3873
17p 3900 fwd	TGGCTGTCCTGAAATACCTATAAA	17p3973-3996
17p 3900 rev	GTGGCCTTTATTTCGGTTTGAA	17p4036-4057
17p 50 fwd	GCCTAGCCAAACATTCCATTT	17p50-70
17p 50 rev	CCCTCATCTAAGTCTTGTCGTT	17p173-179
17p 348 fwd	GGGACAGAAGTGGATAAGCTGATC	17p348-371
17p 348 rev	GATCCCACTGTTTTTATTACTGTTCCT	17p405-431
17q fwd	CACAGAAGAAATGACAATGTGGATT	17q263-288
17q rev	TGGCTTGTGTTTTTGTTCTCTTG	17q326-349
Xq 109 fwd	CCCCTTGCCTTGGGAGAA	Xq109-126
Xq 109 rev	GAAAGCAAAAGCCCCTCTGA	Xq143-162

SupplementaryTable 2: RT-qPCR TERRA Primers	
Name	Sequence
17p TERRA fwd	GGGACAGAAGTGGATAAGCTGATC
17p TERRA rev	GATCCCACTGTTTTTATTACTGTTCCT
7p TERRA fwd	GGAGGCTGAGGCAGGAGAA
7p TERRA rev	CAATCTCGGCTCACCACAATC
Xq TERRA fwd	CCCCTTGCCTTGGGAGAA
Xq TERRA rev	GAAAGCAAAAGCCCCTCTGA
15q TERRA fwd	TGCAACCGGGAAAGATTTTATT
15q TERRA rev	GCGTGGCTTTGGGACAACT
GAPDH Transcript fwd	AGCCACATCGCTCAGACAC
GAPDH Transcript rev	GCCCAATACGACCAAATCC

**Supplementary Table 3: CRISPR Cloning Oligonucleotides**		
Name	Sequence	Target Sequence
17p CRISPR A Top	CACCGTGAACCATGGCGCCACCAGA	Targets 17p3905-3924
17p CRISPR A Bottom	AAACTCTGGTGGCGCCATGGTTCAC	Targets 17p3905-3924
17p CRISPR B Top	CACCGCGCTCTTGCTCACAGTTTCG	Targets 17p3932-3951
17p CRISPR B Bottom	AAACCGAAACTGTGAGCAAGAGCGC	Targets 17p3932-3951
17p Mutant Verif Fwd	TTAGGAGCCACTGTGTCTTGGACG	Targets 17p3632-3655
17p Mutant Verif Rev	GTGGCTGTACCCATTCATGCATTCT	Targets 17p5008-5032

Supplementary Table 4: 17p CIRSPR Homology Block

GAAGCGATGGTGTGGACTAGATCAGTGATAGGGCATGGTGTGGATATTATTACATTAGTATTGGAAGCGATGTTGTGGATT
ACATCAGTGTTAGCGCATGGTGTGAATATTATATAGGTGTTAGGGCACGGTGTGGATATCATAGTAATGTAGAGCACAGTG
TGATTATTATATTAGAGGCCACTGTAAGAATATATATTAGGAGCCACTGTGTCTTGGACGTTGACAATGATATTAGGGTGT
ACTCCAAACAGTGAGATTTGGGGGCTTTATTTTTCTAGATGAATTTCTTCCTCTGCTGAGTGCTCTAAAGACTCACTCCTT
GGCACTCAGGGCCGTGGACAGGAGCTTTTTACTCACCAATGAAGAACACCAAATTAACACGACCCCCGTGCTGCCCTGAGG
AAGCTGAAGCTCCTCGCTGCTTCTGGCACTTCAGCGGGA
-excised 17p3878-3969-
TCCTGGCTGTCCTGAAATACCTATAAAATTCAATATTCAGTTTATTCAGTGTCATAATTTTGGAAATTCAAACCGAAATAA
AGGCCACTATATCCATATCCTTCCCATAAATGTTGATGGAAGAATTATTTGGAAGCCATATAGAATGAAATGACTCTATAC
ACAAATTAAAACACAAAAACCTACTCAAAATAGTCCAGAGACTACAACTTCAAATGCAAAACTATAAATAATCTAACAGAA
AACCTAAGAGACATTCGATCTGGTGTTGAGTTTTAACACACAGCATCAAGTGCCAATTCGCGAAAATACTGAGAACAGACT
TTATAAAACTAAATTTTCTACTATGAAAAACCCTATTCAGAGAACAAAAAGACAAGACACACTGTGAGAAGATATTTACAA
AATACAAACATGATTTTAAAAACTGTATTGAAAATACACAAAGAACTCTTCAAACGAACACTAAGAAAACTAAAAACCCAC
ATAAAACTGGGTAAATATCTGAACAGACATCCAGCCAAATAAAATATATAGATAGCAGGCCAGGTGTGGTGGCTCATGCCT
ATAACCCCAGCACTTTGGGAGGCTGAGGTGGGTGAGTCACCTGAGGTCAGGAATATGAGATCAGCCTGGCCAACATAGTGA
TACCCCCTCTCTACTAAAAATACAAAAAAATTAGCCAGGCATGGTGGTGAGTGCCTGTAATCCCAGCTACTTGGGAGGCTG
ATGCAAGAGAATTGCTTGAACATCGGAGGTGGAGGTTGCAGTGACCCAGGATCACACCACTGCACTCCAGCCTGTGTGACA
GAATGAGACTCTATCTCA
